# Formwork Pressure of a Heavyweight Self-Compacting Concrete Mix

**DOI:** 10.3390/ma14061549

**Published:** 2021-03-22

**Authors:** Michał A. Glinicki, Jacek Gołaszewski, Grzegorz Cygan

**Affiliations:** 1 Institute of Fundamental Technological Research, Polish Academy of Sciences, Pawinskiego Street 5 B, 02-106 Warszawa, Poland; mglinic@ippt.pan.pl; 2 Department of Building Processes and Building Physics, Faculty of Civil Engineering, Silesian University of Technology, Akademicka Street 5, 44-100 Gliwice, Poland; 3 Laboratory of Civil Engineering, Faculty of Civil Engineering, Silesian University of Technology, Boleslawa Krzywoustego Street 7, 44-100 Gliwice, Poland; grzegorz.cygan@polsl.pl

**Keywords:** consistency, formwork pressure, fresh mix, magnetite aggregate, mix design, Portland cement, radiation-shielding concrete, self-compacting concrete, serpentine aggregate, slag cement

## Abstract

High-fluidity and self-compacting concrete (SCC) mixes were developed using special aggregates for radiation-shielding concrete. The special aggregates comprised heavyweight and hydrous aggregates (crushed magnetite, crushed serpentine, and their mixtures), which were selected to provide an enhanced attenuation of gamma and neutron radiation, respectively. For the mixed concrete design with a bulk density of up to 3570 kg/m^3^, two cement types were used: Portland cement CEM I and slag cement CEM III/A. The basic properties of the fresh self-compacting concrete were evaluated and the lateral formwork pressure exerted by the freshly mixed self-compacting concrete was measured and analyzed. An original test setup was developed for the determination of the lateral pressure on the square column formwork with pressure measurements carried out using six strain gauge pressure transducers, which was adequate for heavyweight concrete mixture testing. Self-compacting concrete mixtures containing a magnetite aggregate or blends of serpentine and magnetite aggregates with a slump flow of at least 550 mm were developed. The lateral pressure on the formwork was directly proportional to the density of the self-compacting heavyweight concrete mixes. The maximum values of the lateral pressure recorded in the test at a casting speed of 1.5 m/h did not exceed 27 kPa and 55% of hydrostatic pressure. Concrete mixtures with basalt, magnetite, and magnetite/serpentine blended aggregates were found to develop sufficient shear strength for proper stability during casting.

## 1. Introduction

The performance of radiation-shielding concrete structures that are designed for nuclear power plants is highly influenced by the concrete mix design. Depending on the type of radiation and its energy spectrum, special mineral aggregates are used, such as a high-density aggregate or a hydrogen-bearing aggregate, to enhance the shielding capacity against gamma radiation and neutron radiation, respectively [1,2]. The use of high-density aggregates leads to a heavyweight concrete mix design, usually in the density range of 3000 to 4000 kg/m^3^. A substitution normal-weight aggregate (granite) with a heavyweight aggregate (magnetite) resulted in an initial slump increase, but only had a small impact on the compressive and tensile strengths of the concrete. The design equations of the ACI 349 and International Federation for Structural Concrete (fib) provisions were found useful for predicting the mechanical properties of heavyweight magnetite concrete, except for the modulus of elasticity and the splitting tensile strength [3].

The shielding structure performance may also be affected by inhomogeneities in the concrete placed in the formwork. Voids and flaws in the mix compaction, facilitated by a dense mesh of reinforcing bars and inadequate control of rheological properties, may result in local radiation surges [4,5]. Further important concrete properties, such as the strength and the impermeability of liquids and gases, may also be reduced due to local bleeding, sedimentation, and cold joints as a result of inadequate control of the fresh mix properties. Uncontrolled setting of cement could be a result of using boron-bearing aggregates for enhancing the neutron shielding capacity [6]. Bleeding was found to adversely affect the hardened properties of heavyweight concrete mixes [7]. Every ten years, air permeability tests are run on concrete containment structures to evaluate the actual leakage rate [8] to check whether it is admissible. Cracks and microcracks might significantly increase the permeability of gases and liquids [9]. Laboratory tests on concrete without flaws provide solid evidence of the relationship between concrete density and linear gamma attenuation coefficients, density and mean free path, and density and half- or tenth-value layers of heavyweight concrete (e.g., [10]).

The presented arguments justify seeking methods to master the fresh mix control of concrete that is designed for radiation-shielding structures. Why not aim to use self-compacting concrete (SCC) technology in such a case? A known drawback of SCC technology is the increased formwork pressure that is expected to increase while increasing the density of the concrete; such an effect becomes worse for heavyweight concrete. As is known from the ACI guide [11], the lateral pressure imposed on the formwork is a direct function of the mix density and the rate of the early strength increase. Such effects are incorporated into the maximum pressure calculation of the so-called weight coefficient and chemical coefficient, respectively. More sophisticated models for evaluating the pressure exerted on the formwork by SCC were evaluated on the basis of a round robin test [12] that covered a range of mix densities from 2234–2343 kg/m^3^. The lateral pressure was found to be in the range of 50 to 90% of the hydrostatic pressure for placement rates between 2.7 and 6.4 m/h. The study revealed the general adequacy of the ten examined models for predicting the lateral pressure, pointing out the need for consideration of the structural behavior of concrete at rest.

Apart from the density of concrete and the placement rate, the lateral pressure of an SCC mix on the formwork depends on several factors [13,14,15], including the height of the structural element, the rheological properties of the concrete, the time of starting the concrete strength increase [16,17,18,19], the methods and rate of mixing [16,17,18], the slope and stiffness of the formwork, and the surface smoothness [20]. The research documented in [13,14,15,16,17,18,19,20] shows that through the appropriate selection of casting parameters and rheological properties of the mixture, the pressure on the formwork can be significantly reduced. If the placement rate is lower than 2 m/h and the mixture exhibits a rapid stiffness increase over time, the lateral pressure may not be greater than 50% of the hydrostatic pressure. However, in the DIN 18218 standard [21], it is assumed that when using mixtures with a liquid or self-compacting consistency, almost the full hydrostatic pressure is exerted. A typical vertical formwork used for general construction is designed for the lateral pressure of a concrete mix ranging from 40 to 80 kN/m^2^ and the tensile strength of the ties is 90 to 160 kN. Adjusting the placement rate is completely sufficient for the laying and compacting of normal weight mixes. It has been shown that when concreting walls with ordinary self-compacting concrete at a rate of 2 to 2.5 m/h when it is already at a height of 2 m, the formwork pressure can even take values over 90 kN/m^2^ with braces at 180 kN [22].

In the literature, there is very little data on heavyweight self-compacting concrete. In [23], a self-compacting concrete mix with a density of 3300 kg/m^3^ was developed using a barite aggregate. The obtained slump was 630 mm, the V funnel flow time was 4 s, and the blocking ratio was 0.75. No segregation or visual flaws were observed in columns cast with this mix. Heavyweight concrete mixtures with a high slump of ≥150 mm were developed in [7]; however, the mixtures showed excessive bleeding capacities that ranged from 0 to 11.7% and the bleeding rates ranged from 0 to 5.1 kg/m^2^·h. There is no data available for authors on the formwork pressure of heavyweight self-compacting concrete.

The objective of the current investigation was to develop a design for highly flowable and self-compacting concrete mixes using high-density aggregates and hydrous aggregates, which were selected in order to enhance the shielding capacity against gamma and neutron radiation, respectively. This research also aimed to evaluate the lateral formwork pressure exerted by freshly mixed self-compacting radiation-shielding concrete. The range of the investigation was related to materials that might be useful for the construction of the first Polish nuclear power plant.

## 2. Experimental Program

### 2.1. Materials and Mix Design

The selection of materials for radiation-shielding concrete, especially aggregates, was based on their elemental composition. Elements with a high atomic number provide effective shielding for γ radiation. The attenuation of neutron radiation is enhanced for elements with a low atomic number; therefore, hydrous aggregates containing a large amount of chemically bound water are very effective [24]. Hydrous aggregates stop the release of neutrons through elastic scattering and also absorb lower energy neutrons. Therefore the magnetite and serpentine aggregates, which contained 11.5% bound water, were selected for shielding γ and neutron radiation. Both single-sized coarse aggregates were used (magnetite or serpentine), as well as blends of magnetite with serpentine aggregate and magnetite with the commonly used basalt aggregate. The applied volume ratios of serpentine to magnetite were 2:1 and 1:2 [25]. The selection of cement is important because the kinetics of the lateral pressure decrease is related to the dormant period of cement hydration and the progressive formation of hydration products [18]. A long-term durability-driven selection of cement to reduce the risk of early-age cracking or the deterioration due to alkali–silica reactions or sulfate attack resulted in two types of cement being selected, namely, Portland cement CEM I 42.5 N and slag cement CEM III/A 42.5N, conforming to BS EN 197-1 [26], which provided special properties including LH (low hydration heat), NA (low alkali content) and HSR/SR3 (sulfate resistance) properties [27].

The properties of both types of cement are presented in Table 1 and Table 2, while the properties of the crushed aggregates are presented in Table 3. A polycarboxylate-based superplasticizer high-range water reducer (HRWR) with a specific gravity of 1.1 and solid content of 27% was incorporated into all the mixtures. A high-molecular-weight methylcellulose was employed as the viscosity-modifying admixture (VMA) to enhance the stability, but only for the reference SCC mixtures, not for the special SCC mixtures.

The variable parameters of the self-compacting concrete mix are given in Table 4. The water-to-cement ratio (*w*/*c*) was a constant 0.48, except for one mix, which had a *w*/*c* = 0.60 (but with *w*/*c*_eff_ = 0.48). The aggregate fractions and their amounts were selected to ensure that the mineral grains were properly packed with the minimum amount of natural sand. The principal target properties of the SCC mixes were a slump flow of 600 ± 50 mm and the highest feasible mix density (for gamma attenuation) or the highest feasible content of hydrogen in the aggregates (for neutron attenuation). For such a consistency, a lower initial lateral pressure and faster rates of pressure drop with time could be expected [19]. For the attenuation of the mixed radiation, a blend of high-density aggregate and hydrous aggregate was targeted. The composition was established after a series of trial mixes. The composition of the self-compacting concrete mixtures is shown in Table 5 (the mixture coding is presented in Table 4).

The serpentine aggregate was characterized by high water absorption (Table 3). Therefore, several concrete mixes were designed with either a dry or prewetted serpentine aggregate or with the replacement of all or part of the 0–2 mm serpentine fraction with 0–2 mm quartz sand. The mixtures were designed for compaction by vibration, not as SCC mixtures, to cover the effects of the serpentine aggregate in more detail via the replacement of the 0–2 mm fraction with quartz sand or by using water-saturated aggregate grains. The composition of these mixtures is given in Table 6 (the mixture coding is presented in Table 4).

### 2.2. Test Methods

**Figure 1:**
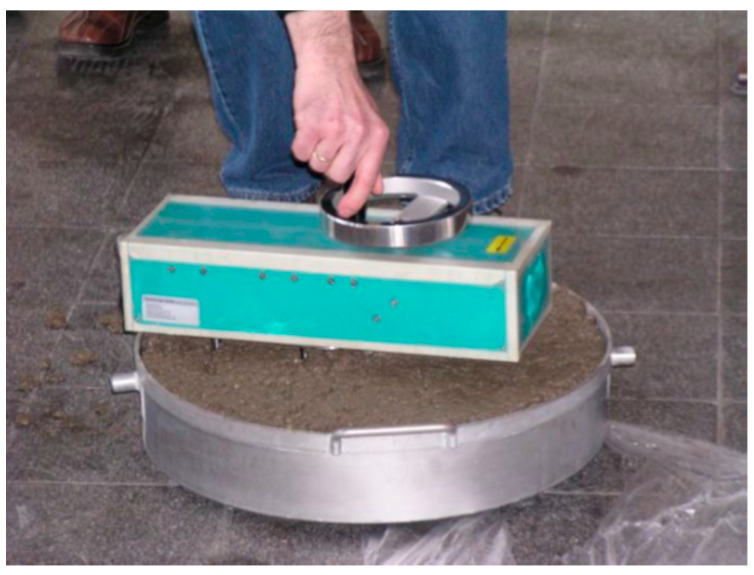
BT2 rheometer in use for the rheological properties determination.

The concrete mixes were prepared in a 30 dm^3^ planetary pan forced mixer. The following properties of the mixtures were determined:

- The mix consistency using the slump method BS EN 12350-2 [30] or the spreading method according to BS EN 12350-8 [31];
- The air content and the mix density according to BS EN 12350-7 [32] and BS EN 12350-6 [33], respectively;
- The resistance to segregation of the mixture according to BS EN 12350-11 [34];
- The rheological properties using a BT2 rheometer (Teubert u. Greim GmbH, Buchbach, Germany) (Figure 1).

For the measurement, the BT2 rheometer was placed in the middle of the container filled with the concrete mixture and then one full revolution was made. The tests adopted a constant measurement time (full rotation) of 15 ± 2 s. During this rotation, the torque and angular velocity were measured with two probes. On this basis, the rheological parameters of the mixture were calculated according to the Bingham model, namely, the yield stress *g* and the plastic viscosity *h*; however, they are presented in conventional units, not physical units. Using measurement constants, one may represent the values of the yield stress and the plastic viscosity in physical units, but the measurement constants have not yet been determined for the BT2 rheometer. The general principles for measuring parameters with the BT2 rheometer are presented and discussed in detail in [35]. Three measurements of the rheological parameters were made for each concrete mix tested.

For the determination of the lateral pressure on the formwork, the mixture was placed in the square column formwork 20 min after mixing. The formwork was filled up to a height of 1 m at a rate of 1.5 m/h. After reaching this height, further concrete placement was simulated by placing 25 kg weights (up to eight pieces) at appropriate time intervals. The load was transferred by means of a 20 cm × 20 cm mold insert, which was placed on the surface of the mixture. One weight represented the load of a concrete mix layer that was 20 ± 5 cm thick, depending on the mix density. Thus, in order to obtain a load corresponding to the concreting speed of 1.5 m/h, weights were laid about every 8 min. This method corresponded to the method of laying the mixture in the formwork in subsequent layers. However, it should be remembered that this method of loading does not take into account the influence of the adhesion of the mixture to the formwork. Thus, the resulting pressure on the formwork may be greater than in reality.

**Figure 2:**
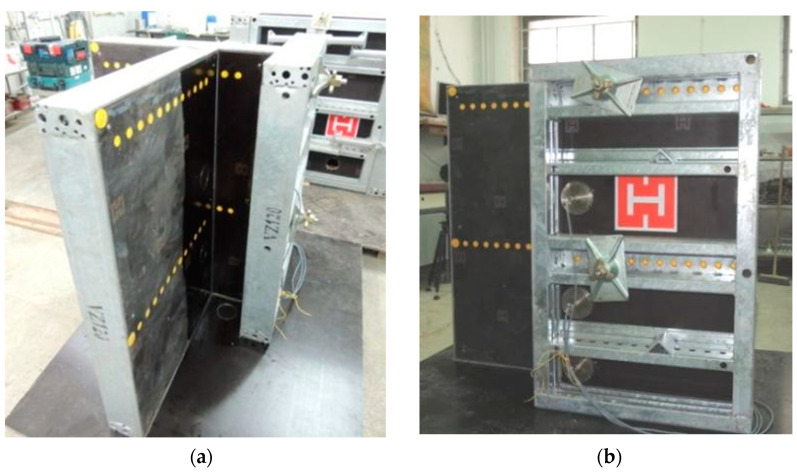

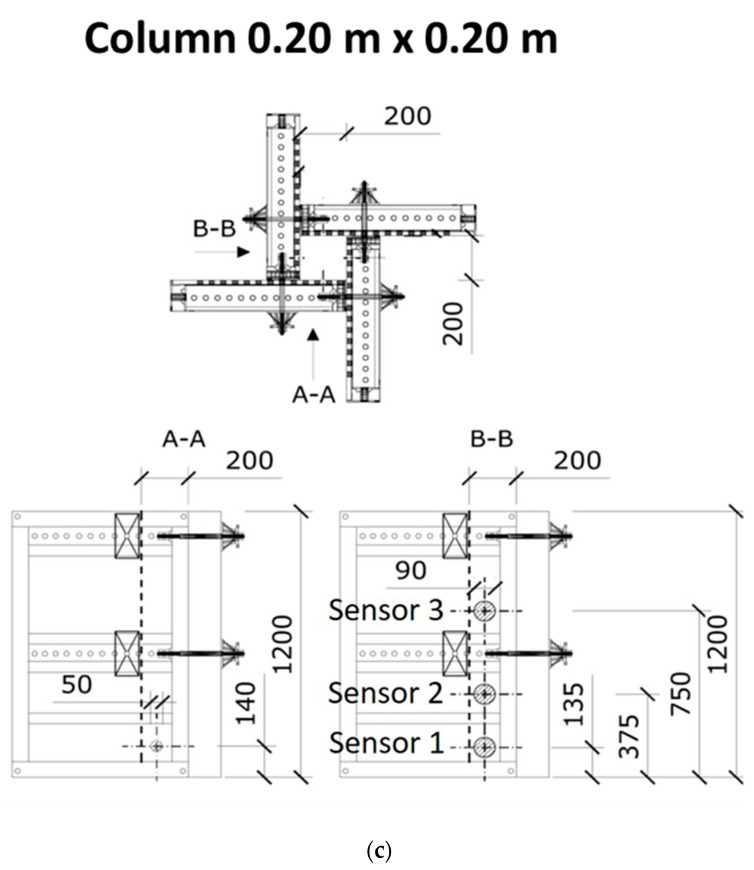
The formwork that was instrumented for the measurements of lateral pressure exerted by the heavyweight concrete mixtures (size in mm): general view of formwork (**a**,**b**) and formwork dimensions and sensor locations (**c**).

The pressure measurement was carried out using six strain gauge pressure transducers located on two opposite formwork walls. Such a strain gauge sensor method has previously been used to measure the formwork pressure [36]. The sensors were placed at 135 mm, 375 mm, and 750 mm from the bottom of the column (Figure 2). These distances refer to the center of the measuring diaphragm, which was a circle with a diameter of 82 mm.

Companion tests were performed to establish the hardened concrete properties. The compressive strength was tested on 150 mm cube specimens after 28 days of standard moist curing according to BS EN 12390-3 [37]. The depth of the water penetration was determined on 150 mm cubes cut out of the columns at the age of two years following BS EN 12390-8 [38]. The cubes were cut from the top and bottom parts of a column. The compressive strengths of the concrete in the columns were also tested. The measurement of the water penetration was important to provide an estimation of the watertightness of the concrete and evaluate the possible effects of the mixture segregation.

## 3. Test Results

### 3.1. Rheological Properties of the Self-Compacting Concrete

The properties of the fresh self-compacting concrete made of CEM I and CEM III/A cement are presented in Table 7. Regardless of the cement type, by using a properly selected amount of a superplasticizer, we obtained mixes with CEM I or CEM III/A with magnetite aggregate (S1B1, S3B1) or with magnetite and serpentine aggregates (S1B3, S1B4) with a target SF1 consistency class (according to BS-EN 206 [39]). The slump flow was within the range of 550–640 mm. The highest slump flow was characteristic for reference mixes (S1B0, S3B0) and it was well maintained over time. Maintaining such a flowability over time was problematic in the case of a larger amount of serpentine aggregate. The maximum density of the mixes with the magnetite aggregate was 3568 kg/m^3^. For blends of magnetite and serpentine aggregates, the bound water content in the aggregates was up to 4% of the concrete mass, which is important for gamma and neutron radiation shielding [24]. The air content in the mixes was mostly in the range from 2.6 to 5%. All the tested radiation-shielding concrete mixtures did not tend to segregate and were usually more stable than those of the control mixtures. The segregation resistances of all the mixes were classified as SR2 according to [39]. The effects of the special aggregates on the yield stress and plastic viscosity were significant, where a relative increase of both properties was found for mixes with magnetite and serpentine aggregate in relation to the reference mixes.

### 3.2. Lateral Pressure on the Formwork

**Figure 3:**
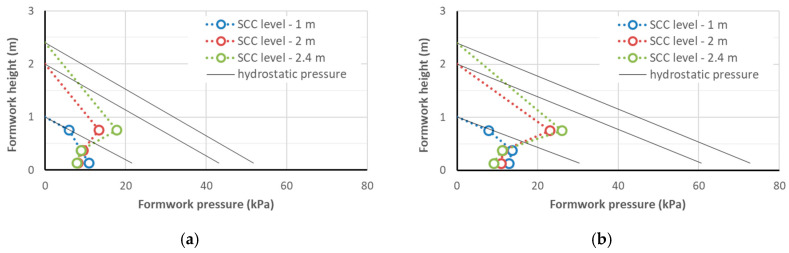
The lateral pressure on the formworks of CEM I self-compacting concrete (SCC): mix S1B0 (**a**) and S1B1 (**b**).

**Figure 4:**
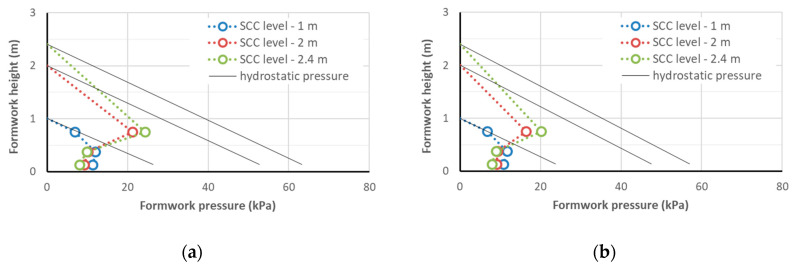
The lateral pressure on the formworks of CEM I self-compacting concrete: mix S1B3d (**a**) and S1B4d (**b**).

**Figure 5:**
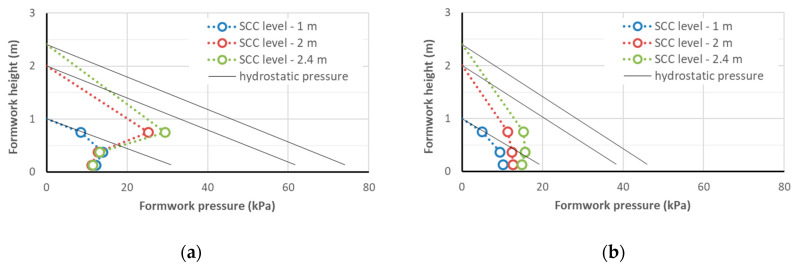

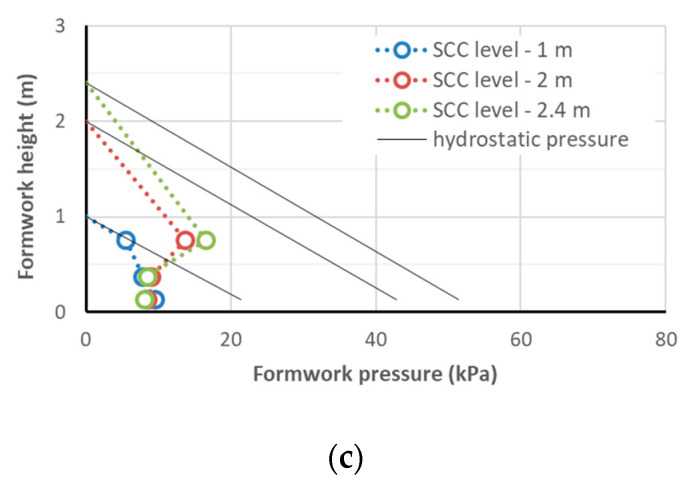
The lateral pressure on the formworks of CEM III self-compacting concrete: mix S3B1 (**a**), S3B2m (**b**), and S3B0 (**c**).

The results of the measurements of the lateral pressure exerted on the formwork by the SCC mixtures are given in Table 8. The maximum lateral pressure on the formwork obtained at the concreting speed of 1.5 m/h that was measured at the moment of reaching the heights of the concreted column of 1 m, 2 m, and 2.4 m (i.e., after 40, 80, and approximately 95 min, respectively) is shown in Figure 3, Figure 4 and Figure 5. With the same SF1 consistency class, the formwork pressure of the radiation-shielding concrete with a magnetite aggregate was clearly larger than the control mix with basalt aggregate; the pressure recorded for the concrete containing only the serpentine aggregate was similar to the control one. However, the formwork pressure did not exceed 27 kPa, which, given the strength of typical formwork at the level of 80 kPa and the working load of typical ties being >90 kN, did not carry a danger of overloading the formwork. It should be noted that the pressure on the column’s formwork was studied with a small cross-section and, as research shows [40], in the case of wall concreting, it can even be twice as high. However, even in such an extreme case, if a concreting speed of not more than 1.5 m/h is maintained, SCC concreting can be carried out safely in typical formwork.

### 3.3. Hardened Concrete Properties in the Columns

The results of the companion tests of the hardened concrete properties are presented in Table 9 as the average value from three cube specimens cut out of cast columns. For most concrete series with magnetite and serpentine aggregates, the compressive strength was within the range of 58–75 MPa, where the highest strength values were recorded for CEM III/A cement because of its long-term hardening characteristics. The lowest strength of 56 MPa was attained for the mix with a water-saturated serpentine aggregate with an excessive *w*/*c* ratio. It was higher than the strength of the reference concrete with basalt aggregates. The observed differences in the concrete density for the top and the bottom parts of the column were small, not higher than 1.5%. Therefore, no indication of significant mix segregation was found.

### 3.4. Properties of the Concrete Mixtures with Dry or Water-Saturated Serpentine

The influence of serpentine aggregate and blended magnetite and serpentine aggregate on the workability of fresh concrete and hardened concrete properties are illustrated in Table 10 and Table 11, respectively. The use of only serpentine aggregate allowed for reaching a high content of bound water in the concrete ingredients, from 5.9 to 7.4% by mass of concrete. However, this was largely associated with the poor workability of the fresh concrete. The highest slump after 60 min, in the range from 16 to 20 cm, was revealed for the 0–2 mm fraction replacement by quartz sand or when additional water was added to the mix (V1B2d3 and V1B2d4, respectively). As expected, in the latter case, a significant reduction in the compressive strength was observed. When using the blended special aggregate (magnetite + serpentine), about half the content of bound water in the concrete ingredients could be attained. However, this was associated with quite a good workability of the concrete (a slump of 19–26 cm), which was very well maintained for the CEM I mixtures. The 28-day compressive strengths were in the ranges of 41–53 MPa and 48–56 MPa for the concrete containing serpentine only and blends of serpentine and magnetite, respectively. Only when additional water was added to the mix did the compressive strength of the concrete decrease to 34 MPa. The mixtures with serpentine aggregate exhibited higher air contents than the mixtures with the magnetite aggregate.

## 4. Discussion of the Results

### 4.1. Lateral Pressure on the Formwork

As mentioned in Section 2, the SCC mixes were designed to achieve a 60 ± 5 cm slump flow (SF1 class). The self-compacting mixtures with a magnetite aggregate (S1B1, S1B3d, S1B4d, S3B1) with the same slump flow revealed a higher yield stress than the control mixtures. This was due to the difference in the densities of these mixtures and the density’s effect on the slump flow: at a given yield stress, the slump flow of the higher density mixture was larger due to the higher weight of the cone. The plastic viscosities of the CEM I concrete mixes (S1B1, S1B3d, S1B4d) were significantly higher, nearly 4 times higher than that of the control mixture. Furthermore, in the case of the CEM III/A mixtures, the plastic viscosity was higher, but by only approximately 50%. The increase in plastic viscosity was probably due to the high content of fines of crushed magnetite aggregate. In the case of mixtures with a slump flow of up to 60 cm, the measurement of the flow time *T*_500_ may not be adequate for the assessment of plastic viscosity (e.g., S3B2m flow time = 6.1 s, S3B1 flow time = 5.2 s, S3B2m plastic viscosity = 5.75 N·m·s, S3B1 plastic viscosity = 12.37 N·m·s). The inadequacy of the slump flow test to evaluate the viscosity of the SCC mixtures with a slump flow of 550–600 mm is discussed, e.g., in [40].

### 4.2. Lateral Pressure on the Formwork

**Figure 6:**
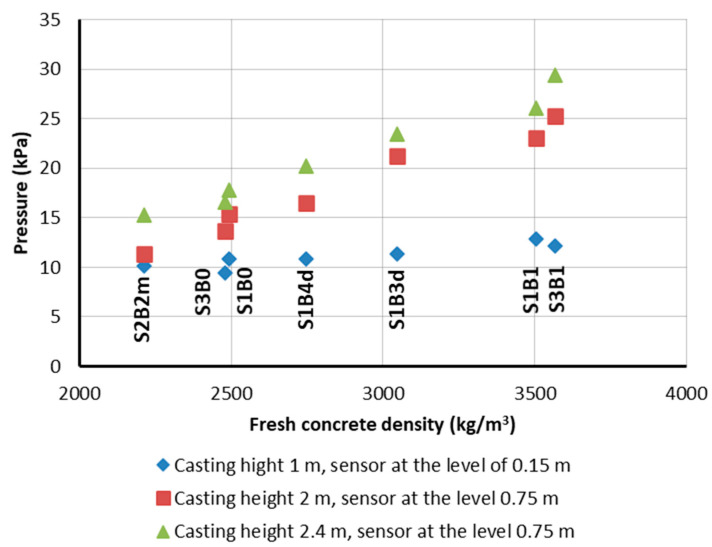
Influence of the fresh concrete density of the SCC on the lateral pressure on the formworks.

**Figure 7:**
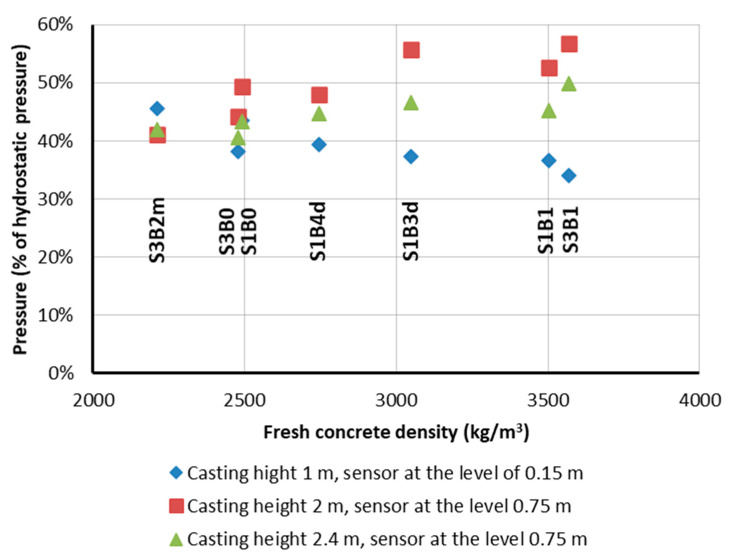
Relative lateral pressure on the formworks (with respect to the hydrostatic pressure) of the fresh SCC with different densities.

The maximum lateral pressure of the self-compacting concrete mixtures with a similar slump flow (consistency class SF1, slump flow from 55 to 65 cm) on the formwork of the column with a cross-section of 0.20 m × 0.20 m was directly proportional to their density (Figure 6). At the same time, it should be noted that the relative pressures on the formwork of the tested mixtures, expressed as a percentage of the hydrostatic pressure, were very similar (Figure 7). This indicates that the relationships for the normal concrete that can be used to predict the pressure on the formwork can also be used for radiation-shielding concrete mixes, after considering their density. It is possible to observe the beneficial effect of the increased plastic viscosity of the mixture on the formwork pressure: the viscosity of the S1B3d and S1B4d mixtures with a slump flow of 55 cm was about twice as high as for the S3B2m mixture. Therefore, the relative pressure on the formwork of these mixtures at the height of 1 m was lower (45 and 55% of the hydrostatic pressure, respectively). This can be explained by the greater adhesion of the more viscous mixture for the formwork, as a result of which, the vertical load was reduced. However, the effect of the viscosity may be less important for elements with a larger cross-section. A lack of segregation of the self-compacting mixtures placed in the columns is also addressed in Section 4.3, which is devoted to the properties of hardened concrete in the columns.

For all the tested mixtures, with the exception of the mixture with serpentine S3B2m, the maximum lateral pressure was recorded for the mixture column height of 2.4 m with the sensor at the 0.75 m level. This pressure did not exceed 27 kPa and was about 50–55% of the hydrostatic pressure. At this point, the formwork pressure at the level of the lower sensors was 2–3 times smaller and showed a tendency to decrease despite the increased load. This means that at the concreting speed of 1.5 m/h, the level of the highest pressure on the formwork was about 1.5 m below the level of the concreting; at greater depths, the pressure on the formwork did not increase and even decreased despite the progress of the concreting. When the concrete mix was left at rest in the formworks, it developed shear strength, which increased with time. The presence of high-density aggregates and large aggregate grains in the self-compacting mixtures (grain size up to 16 mm), as well as in a small volume of paste, favored the faster build-up of a consolidated internal structure and developed a shear strength that was capable of carrying vertical loads. The decrease in pressure was the result of the development of shear strength due to the disappearance of the superplasticizer effect and the progressing process of cement hydration. It should be kept in mind that if the casting rate is increased, the specified concrete height is achieved in a shorter time. The time of resting of the mixture is also reduced, and thus, its shear strength is lower [40,41]. Therefore a higher casting rate results in higher lateral pressure.

In the case of the mixture with the serpentine aggregate, namely, S3B2m, the pressure distribution on the formwork was different from the others. In this case, the pressure at the levels of all the sensors was quite similar. After the end of casting in the formwork, the S3B2m mixture did not obtain the ability to carry as much of a load as the other mixtures: the pressure at the bottom of the formwork was higher than for the other mixtures, despite having the lowest mix density. On the basis of this investigation, such an effect cannot be explained.

Relating the results obtained to previous studies, it can be stated that no systematic test results on the formwork pressure of SCC designed for radiation-shielding have been published [40]. It is unknown whether the features of the pressure exerted on the formwork by a plain SCC and a radiation-shielding SCC are the same. The concretes used in the study were designed such that the cement paste content was as low as possible, whereas for plain concrete, the cement paste content is just sufficient for the high fluidity of mixtures. This low cement paste content was a condition for high resistance to mix segregation and for an enhanced vertical load-carrying capacity to be achieved soon after concreting [40,42]. Due to the low paste content, it was possible to avoid mineral additions or stabilizing admixtures (except for the reference mixes S1B0, S3B0), and obtain a simple mix design that is less prone to variable technological factors and easier to be produced [43]. It should be noted that the content of the fines of the crushed magnetite aggregate, which increased the density of the paste, was beneficial for the mix stabilization. A high water absorption characteristic for the serpentine aggregate (see Table 3) impaired the high fluidity of the mix and accelerated the loss of workability. To avoid such negative effects, some methods that are known in the case of a lightweight aggregate are applicable: water-soaking of the aggregate or a reduction of the fine aggregate content (see Section 3.4 and Section 4.4).

In the investigation reported in [44], the lateral pressures of the column formworks that were exerted by different SCCs with gravel aggregates were investigated. The methodology of the study was analogous to that presented in Section 2.2. Self-compacting mixtures with CEM I and CEM III and *w*/*c* = 0.3 and 0.4 were used. The slump flows of these mixtures were in the range of 600 to 750 mm and the casting rate was 1.0 m/h. The maximum pressure on the formwork obtained after the end of casting at a height of 1 m was in the range of 9 to 14 kPa, and at a height of 2.4 m, the maximum pressure was in the range from 12 to 23 kPa. A comparison of the results of the current investigation and those from [44] revealed that despite the greatly increased mix density, the formwork pressures of normal and shielding concrete were at a similar level. Such a beneficial effect can be attributed to the mix design with a small paste content, which promoted rapid development of the load-bearing capacity of the aggregate skeleton in the concrete [40].

Referring to the current state of the knowledge [40,42,45,46,47,48,49], the characteristic features of formwork pressure of radiation-shielding SCC seem to be similar to those of SCC with common rock aggregates. Therefore, it is reasonable to assume that the formwork pressure models presented in [12] could be applicable for the lateral pressure prediction of SCC with a special heavyweight or hydrous aggregate. As expected, the pressure was proportional to the mix density, but at each concreting stage, it did not exceed 60% of the hydrostatic pressure. This is an important observation because the use of an internal vibrator during the casting of plain concrete mix can lead to a full hydrostatic formwork pressure. Casting walls with a typical rate not greater than 1 to 1.5 m/h with an SCC mix was found to be much safer to perform.

### 4.3. Hardened Concrete Properties in the Columns

In [50], significant differences in the permeability and moisture diffusion coefficient of heavyweight concrete were found, depending on the location of the core drilling specimens and mix design of the concrete. Therefore, after concrete hardening in the formworks, the properties of concrete were established to reveal evidence of segregation, if any. The results summarized in Table 9 show no significant difference in the concrete density with CEM I in the specimens cut from the bottom and top of the column. It indicates a lack of segregation of concrete. Although for two out of three mixes with CEM III/A cement, the difference in the top and bottom density was increased, the difference was still not significant enough to indicate mix segregation.

A greater depth of water penetration in the direction parallel to concreting than in the perpendicular direction was found. An explanation of this effect could be a vertically directed capillary pore system resulting from bleeding and the orientation of the interfacial transition zone relative to the casting direction. However, this issue requires further clarification. The water penetration resistance of the concrete made of CEM III/A cement in the direction perpendicular to concreting was higher than that of concrete made of CEM I cement. This was expected due to the densification of the hydrated paste and the interfacial transition zone, which is associated with long-term hydration of this cement type. In the parallel direction, the beneficial effect of using CEM III/A cement cannot be seen. Increasing the amount of serpentine aggregate in CEM I concrete led to a significant increase in water penetration; in the case of S1B4d concrete, complete water penetration through the specimen in both directions after exposure to water pressure was observed. Interestingly, quite low water penetration was revealed in the concrete with CEM III/A and serpentine aggregate. The observed differences in the compressive strength of the concrete in cut specimens were relevant to the different rates of hardening associated with the types of cement used.

### 4.4. Properties of Concrete Mixtures with Dry or Water-Saturated Serpentine

Poor concrete properties with serpentine aggregate were reported in [5], indicating a low mechanical strength of the aggregate as the main reason for the low concrete strength, but no systematic data was published in regard to the workability and strength of concrete containing dry or presaturated serpentine aggregate. The data presented in Table 10 and Table 11 are related to concrete mixtures that were compacted by vibration. The presence of serpentine, both dry and saturated with water, in mixtures containing both magnetite and serpentine aggregate, made it difficult to obtain consistency class S4 (V1B3, V3B3, V1B4, and V3B4). To increase the content of the serpentine aggregate, it was necessary to increase the content of the superplasticizer, much more than in the control mix (see Table 6). Despite the higher amount of superplasticizer, these mixtures usually lost workability faster.

Despite a high superplasticizer content, it was impossible to obtain an S4 consistency at a *w*/*c* ratio of 0.48 when using dry serpentine aggregate. In order to obtain the required consistency in such a case, it was necessary to significantly increase the amount of water added to the mixture (V1B2d4). However, this induced a marked reduction in the compressive strength of the concrete. A more efficient way to obtain an S2–S4 mixture consistency was revealed: the use of a presaturated serpentine aggregate (mixtures V1B2m1, V1B2m2, V3B2m3, and V3B2m4). It should be noted, however, that even then, the mixtures sometimes displayed a rapid workability loss (to avoid it, a high superplasticizer addition or its redosing was necessary). Furthermore, the process of aggregate saturation is a technological impediment. The use of serpentine aggregate saturated with water did not adversely affect the compressive strength, which is an important observation. By removing the fraction below 2 mm from serpentine aggregate and replacing this fraction with quartz sand, it was feasible to obtain a stable S4 consistency of the concrete mixture (V1B2d2, V1B2d3) for 60 min without the need for presaturating the aggregate. It should be noted that a partial reduction in the serpentine fraction below 2 mm (V1B2d2) did not bring any significant improvement in the mixture’s workability.

It should be noted that the use of the magnetite aggregate allowed for good workability of the mixtures (V1B1, V1B3) to attain the consistency class S4. The amount of superplasticizer needed to obtain the slump of 20 cm was the same for mixtures with magnetite (V1B1) as for the control mixture (V1B0); these mixtures also did not differ significantly in terms of the workability loss.

## 5. Conclusions

The following conclusions can be drawn from the performed investigation:

1. Heavyweight concrete mixes with a density of up to 3570 kg/m^3^ and mixtures with a chemically bound water content of 3–7% with self-compacting characteristics related to a slump flow from 55 to 65 cm were obtained using crushed magnetite and serpentine aggregates. A stable consistency was obtained for the magnetite aggregate, while the presence of serpentine aggregate resulted in a loss of slump flow by 10 cm. Both the yield stress and plastic viscosity of the self-compacting mixtures were increased with the use of magnetite and serpentine aggregates. The mixes exhibited resistance to the segregation of the SR2 class.
2. An original test setup was developed for the determination of lateral pressure on the square column formwork with pressure measurements carried out using six strain gauge pressure transducers located on two opposite formwork walls. The setup was found to be adequate for heavyweight concrete mixtures when testing up to the density of 3570 kg/m^3^.
3. Similar to conventional concrete, at the same casting rate and mix consistency, the maximum lateral pressure was directly proportional to the density of the self-compacting radiation-shielding concrete mixes. The maximum values of the lateral pressure recorded in the test at a casting speed of 1.5 m/h did not exceed 27 kPa and 55% of the hydrostatic pressure. Concrete mixtures with basalt, magnetite, and magnetite/serpentine blended aggregates were able to develop shear strength after casting for proper stability during concreting.
4. At the casting speed of 1.5 m/h, the maximum pressure was registered 1.5 m below the concreting level. The pressure on the formwork was changed or even decreased at deeper levels, despite the progress of concreting. The mixture with the serpentine aggregate showed a lower load-carrying capacity, where in this case, despite the lowest density, the highest pressure on the formwork was observed at the level of the bottom sensor.
5. The dependencies determined for normal concrete that allow for the prediction of pressure on the formwork can also be used for radiation-shielding concrete after taking into consideration their different densities.
6. No flowable concrete mixes with a dry serpentine aggregate were obtained. For a presaturated serpentine aggregate with water in the amount corresponding to its water absorption, concrete mixtures with an S3–S4 consistency could be obtained while avoiding a significant reduction in the strength of the concrete. For proper workability control, it is more beneficial to replace the 0–2 mm fraction of the serpentine aggregate with ordinary quartz sand.
7. It is possible to obtain concrete mixtures with magnetite aggregate with an S4 consistency S4 that is stable over time and mixtures with serpentine and magnetite aggregates blended in various proportions with an S3–S4 consistency. For a higher dose of superplasticizer, an increased loss of workability was observed, which was more pronounced for CEM III/A cement than for CEM I cement.

## Figures and Tables

**Table 1:** Chemical compositions of both types of cement (%), which were determined as per BS EN 196-2 [28].

Component	CEM I 42.5N LH/SR3/NA	CEM III/A 42.5N LH/HSR/NA
SiO_2_	21.48	31.38
Al_2_O_3_	4.80	5.98
Fe_2_O_3_	2.62	2.09
CaO	65.60	52.51
MgO	0.87	3.73
SO_3_	2.84	1.45
K_2_O	0.47	0.56
Na_2_O	0.12	0.34
Cl	0.008	0.058
Loss on ignition	1.12	0.12

Special properties of cement: LH—low hydration heat, NA—low alkali content and HSR/SR—sulfate resistance.

**Table 2:** Physical properties and the strength of both types of cement as per BS EN 197-1 [26].

**Cement Designation**	**Flow (cm)**	**Le-Cha Soundness (mm)**	**Water Demand (%)**			**Setting Time (min)**			
						**Initial**		**Final**	
CEM I 42.5N LH/SR3/NA	18.1	1	28.0			185		250	
CEM III/A 42.5N LH/HSR/NA	15.4	0	34.0			200		345	
**Cement Designation**	**Blaine (cm^2^/g)**	**Density (g/cm^3^)**	**Bending Strength at Days (MPa)**			**Compressive Strength at Days (MPa)**			
			**2**	**7**	**28**	**2**	**7**		**28**
CEM I 42.5N LH/SR3/NA	3800	3.15	3.6	6.0	8.1	20.7	33.7		52.6
CEM III/A 42.5N LH/HSR/NA	4700	2.99	3.0	5.7	9.5	14.2	29.6		58.2

**Table 3:** The properties of the crushed rock aggregates as per BS EN 1097 [29].

Type of Aggregate	Density (kg/dm^3^)	Water Absorption (%)
Crushed basalt	3.0	0.80
Crushed serpentine	2.60	0/2 mm: 2.14 2/8 mm: 2.41 8/16 mm: 1.47
Crushed magnetite	4.80	0.40

**Table 4:** Mixture coding.

Concrete	Cement	Aggregate
S—self-compacting concrete V—vibrated concrete	1—concrete with CEM I 3—concrete with CEM III/A	B0—basalt + magnetite B1—magnetite B2—serpentine B3—serpentine + magnetite 2:1 B4—serpentine + magnetite 1:2 d—dry serpentine aggregate m—water-saturated serpentine aggregate

**Table 5:** Mix design of the self-compacting concrete with high-density and hydrous aggregates (kg/m^3^).

Mix Constituents, *w*/*c* Ratio		Concrete Mix				
		S1B0 S3B0	S1B1 S3B1	S3B2m (1)	S1B3d (2)	S1B4d (2)
Cement (CEM I or CEM III)		350	350	350	350	350
Water		168	168	211	168	168
*w*/*c*		0.48	0.48	0.60	0.48	0.48
*w*/*c*_eff_		-	-	0.48	-	-
Quartz sand 0/2		687	371	371	371	371
Crushed basalt 2/16		1001				
Crushed magnetite 0/5		300	839		772	895
Crushed magnetite 0/16			1846		1018	
Crushed serpentine 0/2				273		
Crushed serpentine 2/8				909	485	485
Crushed serpentine 8/16				273	371	485
HRWR (% mass of cement (%m.c.))	CEM I CEM III/A	0.36 0.2	0.3 0.2	1.4	2	1.6
VMA (%m.c.)	CEM I	0.15				
Designed mix density		2506	3574	2389	3537	2756

(1) Water-soaked serpentine aggregate—3% by weight; water content in the mixture was not corrected. (2) Dry serpentine aggregate. VMA—viscosity-modifying admixture (methylcellulose), HRWR—(polycarboxylate-based) high-range water reducer.

**Table 6:** Mix design of the concrete (kg/m^3^) containing crushed magnetite and/or crushed serpentine in the dry or prewetted state that was compacted using vibration.

Mix Constituents, *w*/*c* Ratio		Concrete													
		V1B0 V3B0	V1B1 V3B1	V1B2d1	V1B2d2	V1B2d3	V1B2d4	V1B2m1	V1B2m2	V3B2m3	V3B2m4	V1B3m	V1B3d V3B3d	V1B4m	V1B4d V3B4d
Cement (CEM I or CEM III)		350	350	350	350	350	350	350	350	350	350	350	350	350	350
Water		168	168	168	168	168	211	211	168	200	200	189	168	189	168
*w*/*c*		0.48	0.48	0.48	0.48	0.48	0.60	0.60	0.48	0.57	0.57	0.54	0.48	0.54	0.48
*w*/*c*_eff_		-	-	0.35	0.46	0.47	0.48	0.48	0.35	0.48	0.48	0.48	-	0.48	-
Quartz sand 0/2		687	371	371	510	654	371	371	371	371	371	371	371	371	371
Crushed basalt 2/16		1001													
Crushed magnetite 0/5		300	839									772	772	895	895
Crushed magnetite 0/16			1846									1018	1018		
Crushed serpentine 0/2				273	136		273	273	273	273	273				
Crushed serpentine 2/8				909	909	909	909	909	909	909	909	485	485	485	485
Crushed serpentine 8/16				273	273	273	273	273	273	273	273	371	371	485	485
HRWR (%m.c.)	CEM I CEM III/A	0.2 0.15	0.2 0.15	1.2	1.5	1.0	0.5	0.5	2.0	0.5	1.66	0.3	0.3 0.2	0.48	0.57 0.4
Remarks					(1)	(2)	(3)	(4)	(5)	(6)	(6)	(6)		(6)	

Remarks: (1) 50% fraction of 0–2 mm of serpentine replaced with quartz sand (by volume). (2) 100% fraction of 0–2 mm of serpentine replaced with quartz sand (by volume). (3) Additional water added to the mixture (3% of serpentine by weight). (4) Water-saturated serpentine: water content of 3% of serpentine by weight. (5) Water-saturated serpentine: water content of 3% of serpentine by weight, content of water in the mixture was corrected. (6) Water-saturated serpentine: water content according to Table 3.

**Table 7:** Properties of the fresh self-compacting concrete used in the determination of the lateral pressure on the formworks.

Property		Cement CEM I				Cement CEM III		
		S1B0	S1B1	S1B3d	S1B4d	S3B0	S3B1	S3B2m
Slump flow (cm)	5 min	64	58	60	55	63	61.5	55
	20 min	62	52	50	43	58.5	53	42
Flow time *T*_500_ (s)	5 min	3.1	5.3	5.5	7	3.4	5.2	6.1
	20 min	4.6	13	11.6	-	6.9	5.3	-
Yield stress *g* (N·m)	5 min	0.35	1.18	0.95	1.12	0.36	1.08	0.53
	20 min	0.42	1.46	1.09	1.86	0.59	1.79	1.06
Plastic viscosity *h* (N·m·s)	5 min	2.7	11.19	11.79	10.46	8.54	12.37	5.75
	20 min	4.62	16.09	13.27	14.63	9.58	12.65	8.98
Air content *A*_c_ (%)		3.8	5	3.8	3.0	3.6	2.6	6.8
Density of fresh concrete (kg/m^3^)		2448	3503	3048	2745	2478	3568	2213
Segregation resistance (SR)		11.2	1.4	6.4	1.8	5.3	3.4	8.9
SR class		SR 2						

**Table 8:** Lateral pressure on formworks.

Mixture	Density (kg/m^3^)	Level of the Sensor (m)	Pressure (kPa) after the End of Casting at a Height of		
			1 m	2 m	2.4 m
S1B0	2448	0.75	6	15.35	17.8
		0.375	8.85	9.35	8.9
		0.135	9.25	8.15	7.8
S1B1	3503	0.75	7.85	23.05	26.1
		0.375	13.7	11.35	11.15
		0.135	12.85	10.95	9.05
S1B3	3048	0.75	7	21.2	24.4
		0.375	12	10.1	9.85
		0.135	11.35	9.25	8.1
S1B4	2745	0.75	6.8	16.45	20.25
		0.375	11.65	9.35	9
		0.135	10.8	9.05	7.9
S3B0	2478	0.75	5.45	12.7	15.6
		0.375	7.8	9	8.4
		0.135	8.45	8.5	8.1
S3B1	3568	0.75	8.5	25.3	29.4
		0.375	13.95	12.75	13.1
		0.135	12.15	11.15	11.45
S3B2m	2213	0.75	5.05	11.35	15.3
		0.375	9.35	12.35	15.7
		0.135	10.13	12.65	14.85

**Table 9:** Hardened concrete properties in the columns.

Concrete Mix	Position of Sample in the Column	Density (kg/m^3^)	Average Compressive Strength (MPa)	Depth of Water Penetration (mm)	
				Parallel to Direction of Concreting	Perpendicular to Direction of Concreting
S1B0	Top	2482	63.5	40	50
	Bottom	2468			
S1B1	Top	3595	58.2	60	55
	Bottom	3570			
S1B3	Top	3221	63.8	105	65
	Bottom	3207			
S1B4	Top	2764	55.6	Water penetrated through the sample	
	Bottom	2762			
S3B0	Top	2417	71.5	80	25
	Bottom	2467			
S3B1	Top	3582	75.4	60	45
	Bottom	3536			
S3B2m	Top	2263	61.4	35	20
	Bottom	2287			

**Table 10:** Properties of the fresh and hardened concrete with serpentine aggregate (dry or soaked).

Property		Concrete Mix							
		V1B2d1	V1B2d2	V1B2d3	V1B2d4	V1B2m1	V1B2m2	V3B2m3	V3B2m4
Slump (cm)	5 min	3.0	4.0	22.0	21..5	14	1.0	14	25
	60 min	-	1.0	16.0	16.0	6.0	-	6	20
Air content (%)		2.9	3.2	4.0	4.2	3.0	2.0	3.8	4.0
Density of fresh concrete (kg/m^3^)		2380	2335	2289	2273	2378	2410	2303	2293
Density of concrete (kg/m^3^)		2373	2366	2311	2280	2394	2424	2348	2284
Compressive strength of concrete (MPa)		47.6	52.7	40.7	34.3	47.8	50.5	47.8	46.7

**Table 11:** Properties of the fresh concrete with magnetite and serpentine aggregates (dry or soaked).

Property		Concrete Mix									
		V1B0	V3B0	V1B1	V3B1	V1B3d	V1B3m	V1B4d	V1B4m	V3B3d	V3B4d
Slump (cm)	5 min	20	20	22.0	20	21.0	21.0	19.5	26	19	21
	60 min	19	9	19	4	16	17.5	19.5	21	5	18
Air content (%)		1.9	2.2	2.2	1.8	1.9	2.1	2.4	2.5	2.0	2.8
Density of fresh concrete (kg/m^3^)		2421	2452	3587	3598	3191	3208	2783	2728	3195	2750
Compressive strength of concrete (MPa)		48.0	46.2	54.0	55.3	53.7	48.1	52.6	37.5	-	-

## Data Availability

All necessary data are presented in article.
